# Short-term effects of combining upright and prone positions in patients with ARDS: a prospective randomized study

**DOI:** 10.1186/cc10471

**Published:** 2011-09-29

**Authors:** Oliver Robak, Peter Schellongowski, Andja Bojic, Klaus Laczika, Gottfried J Locker, Thomas Staudinger

**Affiliations:** 1 Department of Internal Medicine I, Intensive Care Unit, Medical University of Vienna, Waehringer Guertel 18-20, A-1090 Vienna, Austria

## Abstract

**Introduction:**

Prone position is known to improve oxygenation in patients with acute lung injury (ALI) and the acute respiratory distress syndrome (ARDS). Supine upright (semirecumbent) position also exerts beneficial effects on gas exchange in this group of patients. We evaluated the effect of combining upright and prone position on oxygenation and respiratory mechanics in patients with ALI or ARDS in a prospective randomized cross-over study.

**Methods:**

After turning them prone from a supine position, we randomized the patients to a prone position or combined prone and upright position. After 2 hours, the position was changed to the other one for another 6 hours. The gas exchange and static compliance of the respiratory system, lungs, and chest wall were assessed in the supine position as well as every hour in the prone position.

**Results:**

Twenty patients were enrolled in the study. The PaO_2_/FiO_2 _ratio improved significantly from the supine to the prone position and further significantly increased with additional upright position. Fourteen (70%) patients were classified as responders to the prone position, whereas 17 (85%) patients responded to the prone plus upright position compared with the supine position (*P *= n.s.). No statistically significant changes were found with respect to compliance.

**Conclusions:**

Combining the prone position with the upright position in patients with ALI or ARDS leads to further improvement of oxygenation.

**Trial registration:**

Clinical Trials No. NCT00753129

## Introduction

Acute lung injury (ALI) and acute respiratory distress syndrome (ARDS) are both frequent conditions in critically ill patients. The incidence ranges from 15 and 34 cases per 100,000 inhabitants per year [1-3]. Compression atelectasis is observed in the dependent parts of the lung, where cardiac weight, abdominal pressure, and pleural effusions compress the lower lobes [4]. Nondependent parts of the lungs are therefore often overventilated and have barotrauma induced by high ventilator pressure [5].

A prone position is known to improve oxygenation in about 70% of patients with ALI or ARDS [6] and to reduce lung stress and strain [7]. A supine upright (semirecumbent) position also significantly improves gas exchange in patients with ALI or ARDS [8,9]. The effect of combining upright and prone positions has not been systematically examined. We hypothesized that in patients with ALI/ARDS, oxygenation improves when combining upright and prone positions because of changes in respiratory mechanics. We therefore conducted a prospective, randomized study to investigate the short-term effects of combined upright and prone positioning on gas exchange and lung mechanics.

## Materials and methods

**Figure 1:**
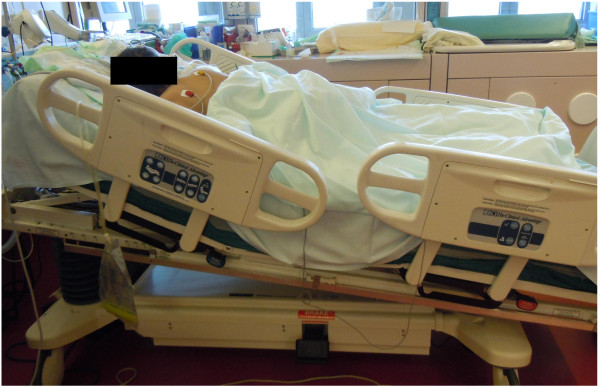
**Example of a patient in prone position with additional upright position achieved by raising the head end and lowering the foot end of the bed**.

**Figure 2:**
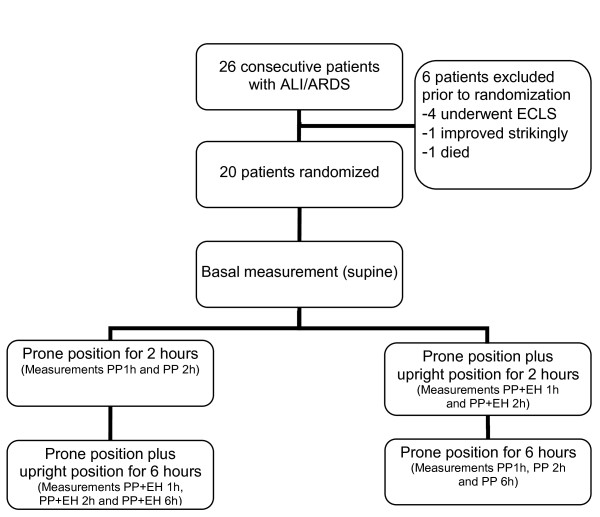
**Flow chart of randomization and study procedures**. ECLS, Extracorporeal lung support.

The study was conducted at a medical intensive care unit of a tertiary care university hospital between October 2008 and April 2010 and was approved by the institutional ethical review board. According to Austrian law, informed consent of unresponsive patients was obtained from them after they regained responsiveness. Patients were eligible for inclusion if ventilated for ALI or ARDS, and the decision to perform the prone position had been taken by the responsible intensivist. ALI and ARDS were defined according to the American-European Consensus Conference [10]. Patients were not eligible for the study if a diagnosis of ARDS had been established more than 3 days before evaluation, if younger than 18 or older than 89 years of age, if pregnant, if severe life-threatening hypoxia (PaO_2_/FiO_2 _ratio <60) was present, or a decision to perform extracorporeal gas exchange was taken. Furthermore, patients with elevated intracranial pressure, elevated intraabdominal pressure, unstable spine fractures, life-threatening arrhythmias, or hemodynamic deterioration in whom prone positioning was contraindicated or at least not advisable were excluded. Randomization was performed by opening sealed envelopes containing the allocation to group A or B. All patients were positioned in a low-airloss bed system before the first proning maneuver (ATP Therapulse; KCI Austria, Vienna, Austria). Prone positioning was performed by turning the patients around the longitudinal axis into complete (180 degree) prone position. The head was positioned by supporting one shoulder with a cushion and turning the head toward one side. Additional upright position was achieved by raising the head end and lowering the foot end of the bed to achieve an angle of at least 20 degrees (Figure 1). Cushions were positioned between the feet of the patient and the end of the bed to guard patients against sliding down. To exclude a time-dependent effect, patients were randomized into two groups: In both groups, basal measurements were performed in a supine position immediately before turning the patient prone; in group A, patients were kept in prone position without an upright position for 2 hours followed by 2 hours of prone-plus-upright position. In group B, patients were placed in an upright position immediately after turning them prone for 2 hours, followed by prone position without upright position for another 2 hours. Every hour, blood-gas analysis and measurement of dynamic and static compliance were performed. After this 4-hour period, patients in group A remained in prone-plus-upright position, and patients in group B in prone position without upright position for another 4 hours. A final assessment of gas exchange was performed (Figure 2). Compliance of the respiratory system was measured with the Bicore Monitoring System (Bicore CP 100, Bicore, Irvine, California, U.S.A.) by using a self-calibrating flow transducer connected to the endotracheal tube (Varflex; Bicore) and a balloon-equipped nasogastric tube to measure esophageal pressure (SmartCath; Bicore). The compliance of the respiratory system was obtained by the occlusion method by using the inspiratory and expiratory hold function of the ventilator (Servo_i_; Maquet, Solna, Sweden). All respiratory-mechanics data were obtained as an average of three measurements. The calculation of chest wall and lung compliance was performed according to [6]. All patients were continuously monitored by ECG, pulse oximetry, and an indwelling arterial catheter. Blood-gas analysis was performed by using an automated blood-gas analyzer (ABL 700; Radiometer Company, Copenhagen, Denmark). Response was defined by an increase in the PaO_2_/FiO_2 _ratio of >10% from baseline. Mechanical ventilation was performed by using a time-cycled pressure-controlled mode. Positive end-expiratory pressure levels were adjusted in increments of 2 cm H_2_O to maintain the FiO_2 _at 0.6 or less with arterial oxygen saturation of >91%, if possible. The inspiration/expiration ratio was set to 1.0 in all patients. Peak inspiratory pressure (PIP) was kept to the lowest possible level to apply tidal volumes of 6 ml/kg ideal body weight. Respiratory rate was chosen to maintain the PaCO_2 _at levels to avoid respiratory acidosis at less than pH 7.25, as well as to avoid dynamic hyperinflation. Ventilator settings remained unchanged during the study period.

### Statistical analysis

The primary end point of the study was a change in the PaO_2_/FiO_2 _ratio. Secondary end points were changes in PaCO_2_, as well as compliance of lung, chest wall, and the respiratory system. Continuous data are given as median and interquartile range. Nonparametric tests were chosen because of the small population studied. To compare groups, the Mann-Whitney *U *test was used for continuous variables. The Fisher Exact test was used to compare dichotomous variables.

To compare the changes in the different positions over time, a nonparametric one-way ANOVA for repeated measures (Friedman test) was used. Dunn's Multiple Comparison post test was used to compare pairs of time points.

Calculations were performed by a statistics software package (GraphPad Prism; GraphPad Software, San Diego, California, U.S.A.). Differences with a *P *level less than 0.05 were considered statistically significant.

### Power analysis

To estimate patient number, a prospective calculation of power was performed during protocol design: Previous trials on the upright position report an increase of the PaO_2_/FiO_2 _ratio between 30 and 40 compared with that in the supine position. Our study was provided with a power of 90% at a two-sided significance level of 0.05 to detect changes in the PaO_2_/FiO_2 _ratio of at least 30.

## Results

Twenty-six consecutive patients with ALI or ARDS were evaluated for inclusion. Six patients were excluded (Figure 2); 20 patients were randomized into the two groups, nine patients into group A, and 11 patients into group B. Patients' demographics and clinical data are presented in Table 1.

**Table 1:** Patient characteristics

	All (*n *= 20)	Group A (*n *= 9)	Group B (*n *= 11)
Gender	7 male	3 male	4 male
	13 female	6 female	7 female
Age (years)	67 (52-74)	68 (55-74)	67 (46-74)
Days on ICU	2.00 (1.25-4.00)	3.00 (1.50-5.00)	2.00 (1.25-4.00)
Days on MV	2.50 (1.00-5.50)	3.00 (1.00-7.00)	2.00 (1.00-3.00)
ARDS/ALI day	2.00 (2.00-3.00)	2.00 (2.00-3.00)	2.00 (2.00-3.00)
Admission SAPS II	52 (37-61)	57 (42-63)	48 (30-60)
LIS	3.00 (2.75-3.50)	3.00 (2.75-3.25)	3.00 (2.75-3.50)
P/F Ratio	138 (118-146)	142 (119-153)	137 (93-146)
PaCO_2_	57 (47-66)	57 (49-69)	57 (49-68)
FiO_2_	0.70 (0.60-0.70)	0.70 (0.60-0.70)	0.70 (0.55-0.90)
Tidal volume (ml)	364 (355-536)	364 (355-562)	364 (353-492)
Peak pressure (cm H_2_O)	30 (29-36)	30 (29-35)	31 (29-36)
PEEP (cm H_2_O)	12 (11-14)	12 (11-17)	12 (10-14)
Respiratory rate	20 (18-20)	20 (17-20)	20 (18-23)
Pulmonary ARDS/ALI	16 (80%)	7 (78%)	9 (82%)
Cause of ARDS/ALI			
Pneumonia	15	7	8
Sepsis	4	2	2
Vasculitis	1		1

No statistically significant differences were found between the groups. Values are expressed as median and IQR unless indicated otherwise. ALI, acute lung injury; ARDS, acute respiratory distress syndrome; ICU, intensive care unit; LIS, lung injury score; MV, mechanical ventilation; SAPS II, simplified acute physiology score.

Fourteen (70%) patients were classified as responders to a prone position, whereas 17 (85%) patients responded to a prone-plus-upright position compared with a supine position (*P *= n.s.). Three patients not responding to a prone position improved only after additional upright positioning. Three patients were classified as nonresponders to either prone position only or an additional upright position. All six nonresponders to a prone position had pulmonary ARDS due to pneumonia. A response to a prone position tended to be associated with lower PaO_2_/FiO_2 _ratio at inclusion (Table 2).

**Table 2:** Univariate comparison between responders and nonresponders to prone position

	Responder (*n *= 14)	Nonresponder (*n *= 6)	*P *value
Age (years)	68 (53-74)	68 (36-83)	ns
Gender	4 male	3 male	ns
	10 female	3 female	
PaO_2_/FiO_2 _ratio	123 (90-149)	169 (13-257)	0.05
PaCO_2_	54 (44-67)	59 (43-70)	ns
SAPS II	60 (37-62)	48 (29-50)	ns
LIS	3.00 (2.75-3.56)	3.00 (2.81-3.50)	ns
C_stat _(inclusion)	23.5 (19.0-28.5)	17.8 (14.0-27.0)	ns
ARDS day	2.00 (1.00-3.00)	2.00 (2.00-3.00)	ns
Pulmonary ARDS/ALI	71%	100%	ns

Values are expressed as median and IQR unless indicated otherwise.

ALI, acute lung injury; ARDS, acute respiratory distress syndrome; LIS, lung injury score;

SAPS II, simplified acute physiology score.

No adverse effects leading to premature termination of the study were observed. All patients remained hemodynamically stable. In all study patients, changes in gas exchange did not necessitate adaptation of ventilator settings.

### Gas exchange

**Figure 3:**
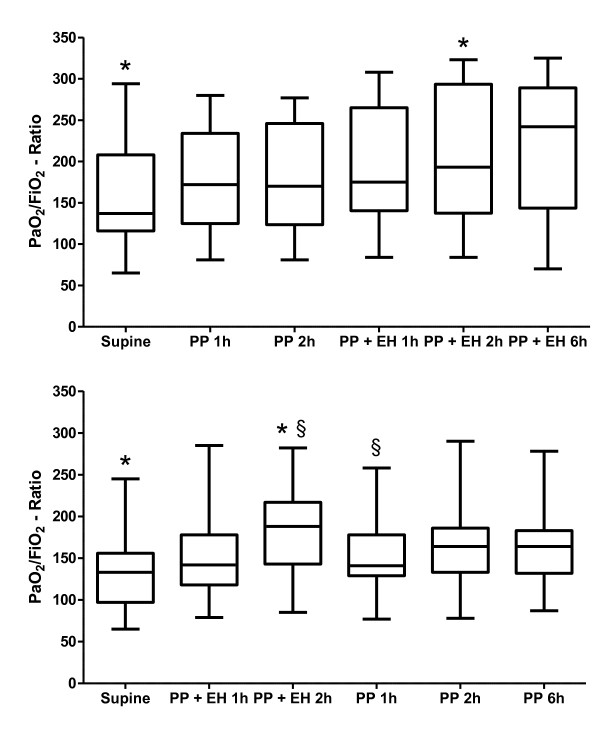
**PaO**_**2**_**/FiO**_**2 **_**ratios of patients in the two randomization groups**. **(a) **Group A: PaO_2_/FiO_2 _ratio of patients at baseline in supine position, after 1 and 2 h of prone position (PP 1 h and PP 2 h), and after 1, 2, and 6 h of prone position with additional upright position (PP + UP 1 h, PP + UP 2 h, and PP + UP 6 h). *Statistically significant increase. **(b) **Group B: PaO_2_/FiO_2 _ratio of patients at baseline in supine position, after 1 and 2 h of prone position with additional upright position (PP + UP 1 h and PP + UP 2 h), and after 1, 2, and 6 h of prone position (PP 1 h, PP 2 h, and PP 6 h). *Statistically significant increase; §statistically significant decrease. PP, prone position; UP, upright position.

The PaO_2_/FiO_2 _ratio improved significantly from supine to prone position and further significantly increased with additional upright position (Table 3, Additional file 1, Figure S1). In group A, oxygenation continuously improved in a prone position, reaching a statistically significant difference compared with the supine position after 2 hours of additional upright position. In group B, the combination of prone and upright position led to a significant increase of oxygenation. This effect was reversed after 2 subsequent hours of prone position without head elevation. After they remained in a prone-plus-upright position for a further 4 hours in group A, we noted a tendency toward a further, yet not statistically significant increase in the PaO_2_/FiO_2 _ratio. In group B, the PaO_2_/FiO_2 _ratio after 4 additional hours in a prone position without an upright position remained at lower levels compared with prone-plus-upright position (Figure 3). The PaCO_2 _did not change significantly during the study period.

**Table 3:** Main results

All patients	Supine	PP 1 h	PP 2 h	PP + UP 1 h	PP + UP 2 h	
PaO_2_/FiO_2_	135 (106-169)^a^	160 (130-185)	165 (136-192)^ab^	160 (118-214)	191 (145-256)^ab^	
PaCO_2_	57 (44-67)	59 (48-66)	58 (48-65)	58 (49-69)	57 (51-69)	
C_tot_	28.0 (22.5-40.2)	30.5 (24.0-36.7)	30.0 (24.2-37.0)	28.5 (24.5-34.5)	28.0 (22.5-34.5)	
C_pulm_	52.0 (37.2-80.0)	48.5 (38.5-72.2)	53.5 (35.7-67.7)	48.0 (37.5-64.5)	48.0 (37.5-66.2)	
C_cw_	76.5 (53.0-104.5)	79.5 (56.0-113.0)	79.0 (55.7-111.0)	84.0 (50.5-105.0)	79.0 (45.2-99.0)	
**Group A**	**Supine**	**PP 1 h**	**PP 2 h**	**PP + UP 1 h**	**PP + UP 2 h**	**PP + UP 6 h**
PaO_2_/FiO_2_	137 (116-208)^c^	172 (125-234)	170 (123-246)	175 (140-265)	193 (137-293)^c^	242 (144-289)
PaCO_2_	62 (50-68)	64 (59-68)	62 (54-68)	65 (56-73)	62 (54-70)	63 (43-75)
C_tot_	28.0 (23.0-38.0)	32.0 (22.0-38.0)	30.0 (17.5-38.0)	30.5 (23.0-36.5)	29.0 (23.5-36.0)	34.0 (24.5-41.5)
C_pulm_	56.0 (45.0-80.0)	63.0 (45.5-81.0)	62.0 (45.0-77.5)	54.0 (48.0-69.5)	55.0 (46.0-74.0)	62.0 (48.0-85.0)
C_cw_	67.0 (39.0-154.0)	65.0 (42.0-121.0)	58.0 (29.0-140.0)	65.0 (42.0-111.5)	59.0 (41.0-116.0)	79.0 (44.0-113.5)
**Group B**	**Supine**	**PP + UP 1h**	**PP + UP 2 h**	**PP 1 h**	**PP 2 h**	**PP 6 h**
PaO_2_/FiO_2_	133 (97-156)^d^	142 (118-178)	188 (143-213)^de^	141 (129-178)^e^	164 (133-186)	164 (132-183)
PaCO_2_	49 (44-68)	56 (46-68)	53 (44-70)	52 (47-67)	50 (47-59)	56 (49-67)
C_tot_	26.0 (22.0-43.0)	28.0 (24.0-33.0)	27.0 (22.0-35.0)	30.0 (24.0-37.0)	31.0 (25.0-37.0)	30.0 (23.0-36.0)
C_pulm_	45.0 (31.0-81.0)	40.0 (33.0-63.0)	43.0 (34.0-67.0)	44.0 (37.0-66.0)	44.0 (33.0-68.0)	44.0 (30.0-56.0)
C_cw_	77.0 (72.0-100.0)	90.0 (68.0-105.0)	82.0 (73.0-91.0)	80.0 (65.0-113.0)	88.0 (71.0-105.0)	82.0 (70.0-140.0)

Values are expressed as median and IQR.

^a^Supine versus PP 2 h; *P *< 0.01; supine versus PP + UP 2 h; *P *< 0.001;

^b^PP 2 h versus PP + UP 2 h; *P *< 0.01.

^c^Supine versus PP + UP 2 h; *p *< 0.05;

^d^Supine versus PP + UP 2 h; *p *< 0.001;

^e^PP+ UP 2 h versus PP 1 h; *P *< 0.05. PP 1 h, PP 2 h, and PP 6 h: 1, 2, and 6 h of prone position. PP+UP 1 h, PP+UP 2 h, and PP+UP 6 h: 1, 2, and 6 h of prone position with additional upright position. C_cw_, compliance (chest wall); C_pulm_, compliance (lung); C_tot_, compliance (total); PP, prone position; UP, upright position.

Analyzing the subgroup of the 16 patients with pulmonary ALI or ARDS due to pneumonia, we observed changes in the PaO_2_/FiO_2 _ratio compared with the whole cohort (Additional file 1, Table S1).

### Respiratory mechanics

No statistically significant changes were found in total respiratory system static compliance, lung static compliance, and chest-wall static compliance comparing the different positions (Table 3). Chest-wall compliance tended to decrease in prone position, yet did not reach statistical significance. When we analyzed responders with respect to oxygenation only, also no statistically significant changes with respect to total respiratory system static compliance, lung static compliance, and chest-wall static compliance were observed (Additional file 1, Table S2).

## Discussion

Our results prove for the first time that combining upright with prone position in patients with ARDS can lead to additional improvement of oxygenation. Prone position exerts an improvement on oxygenation over time up to 12 hours or more [11]. To exclude a time-dependent effect on gas exchange, patients were randomized into two groups alternating the sequence of positioning maneuvers. The deterioration of oxygenation after reversing upright position in group B seems to exclude such a time-dependent effect. In two patients in group A, however, the PaO_2_/FiO_2 _ratio deteriorated after establishing the upright position. Therefore, when turning a patient prone, it seems advisable to evaluate changes in oxygenation before adding the upright position to be able to differentiate between the effects of the methods.

Response with respect to oxygenation could be observed in most of our patients; three patients could be classified as responders only after adding the upright position. Response to proning was associated with a worse oxygenation, indicating a better effect in patients with more-severe ARDS. Eighty percent of our patients had pulmonary ARDS, including all nonresponders. Extrapolating our results to all ARDS patients, including those with extrapulmonary ARDS, therefore seems speculative. As pulmonary ARDS, however, has been reported to be associated with a worse response to upright and prone positioning [8], a comparable or even more-pronounced effect in patients with extrapulmonary ARDS could be possible. Notably, three of six patients with pulmonary ARDS not responding to proning improved only after adding the upright position. Therefore, adding the upright to the prone position seems a simple and advisable measure before classifying patients as nonresponders.

With regard to outcome, the prone position failed to improve outcome significantly in several large, randomized trials [12-14]. A recently published meta-analysis, however, showed favorable effects on survival in patients with severe ARDS with a PaO_2_/FiO_2 _ratio <100 [15]. Most of our patients showed a higher PaO_2_/FiO_2 _ratio at inclusion. Interestingly, a higher response rate with respect to oxygenation was observed in patients with lower PaO_2_/FiO_2 _ratio and a tendency toward higher SAPS II scores, indicating more-severe illness. Obviously, with respect to oxygenation, these patients responded better to positioning maneuvers. Oxygenation has been shown, however, to be a poor surrogate parameter with respect to outcome [16], whereas a decrease in PaCO_2 _after prone position was associated with higher survival rates [17]. Therefore, because of the low number of patients and the very short observation period, no conclusions with respect to any effect on outcome by applying the prone-plus-upright position can be derived from our data. It may be speculated, however, that the additional beneficial effects on gas exchange by modifying the prone position, as in our study, could enable a less-invasive ventilation strategy, thus leading to better survival [18-20].

The mechanisms by which upright position improves oxygenation are not completely clear. Hoste and co-workers [8] observed a significant improvement in oxygenation with the upright position and speculated about a resolution of dorsal atelectases and less pressure of the heart and lung tissue as underlying mechanisms [8]. In this study, no changes in tidal volumes and compliance could be observed. Richard *et al. *[9], reporting a comparable improvement in oxygenation, additionally measured end-expiratory lung volume. Improvement of oxygenation was associated with an increase in end-expiratory lung volume, pointing toward alveolar recruitment or changes in the compliance of the respiratory system [9], whereas in this study in nonresponders, these effects were not observed. When analyzing our subgroup of 16 responders, however, no significant changes in compliance could be observed as well (Additional file 1, Table S2). Recruitment by prone positioning, however, does not necessarily lead to increase of compliance [6]. It has been hypothesized that recruitment by prone position is indicated by a decrease of PaCO_2 _[17]. In the upright-position studies Hoste and co-workers [8] did not observe changes in PaCO_2_, whereas the second study did not report PaCO_2 _values [9]. Thus, as we were not able to detect significant changes in the compliance of lungs and chest wall as well as PaCO_2_, respectively, changes in neither lung volume nor recruitment as possible mechanisms leading to improved oxygenation can be derived from our data. It can therefore only be speculated that a caudal shift of the diaphragm [21] leads to redistribution of ventilation and perfusion, thus optimizing the ventilation-perfusion relation. By turning a patient prone, improvement of the ventilation-perfusion relation is known to be a major factor contributing to improved gas exchange: although resolution of dorsal atelectases leads to better ventilation and recruitment, perfusion is less gravitation dependent and more evenly distributed in the prone than in the supine position, leading to a decrease in ventilation-perfusion mismatch [22,23]. It must be taken into account, however, that in both studies investigating the effects of supine upright position, head-elevation angles between 40 and 45 degrees were reached, whereas in our study, only angles between 20 and 30 degrees could be achieved. During the prone position, head elevation of more than 30 degrees can hardly be reached with standard bed systems, as in the prone position, only a reverse Trendelenburg position (combined with minimal additional elevation of the bed head at the best) can be performed. These lower angles could have led to less-pronounced effects on lung mechanics, more difficult to detect.

It should be noticed that we cannot estimate the long-term effects of combining the upright and prone positions, as our measurements were restricted to an 8-hour period. In the studies investigating the effects of the supine upright position, Richard and co-workers [9] measured short = term effects over a 2-hour period only; in the second study, patients improved over a period of 12 hours [8]. Prone position could be shown to lead to a sustained improvement of oxygenation even after a period of 12 hours [11]. Therefore, sustained beneficial long-term effects on oxygenation when combining both methods may be expected, yet cannot be derived from our data.

## Conclusions

Conclusively, the prone position and the additional upright position may exert additive beneficial effects on oxygenation in patients with ARDS and could be attempted routinely when deciding to turn a patient prone. Upright-prone position is a feasible, easy method to improve oxygenation in patients with ARDS. Strict observation of the effects of each positioning maneuver is mandatory because individual responses may vary greatly.

## Key messages

• Combining prone position and upright position may improve oxygenation in patients with ARDS.

• Individual response to each positioning maneuver varies and makes strict observation mandatory.

## Abbreviations

ALI: acute lung injury; ARDS: acute respiratory distress syndrome; C_cw_: compliance (chest wall); C_pulm_: compliance (lung); C_tot_: compliance (total); ECG: electrocardiography; ICU: intensive care unit; IQR: interquartile range; LIS: lung injury score; MV: mechanical ventilation; PP: prone position; SAPS II: simplified acute physiology score; PIP: peak inspiratory pressure; UP: upright position.

## Competing interests

The authors declare that they have no competing interests.

## Authors' contributions

OR and TS made substantial contributions to conception and design, acquisition of data, analysis and interpretation of data, and writing the manuscript. PS was involved in drafting the manuscript and revising it critically for important intellectual content. KL and GL were involved in drafting the manuscript and revising it critically for important intellectual content and furthermore made contributions to planning the study and statistical analysis. AB made contributions to planning the study and statistical analysis. All authors read and approved the final manuscript for publication.

## Supplementary Material

Additional file 1 **Additional information**. Table S1: Main results (median, IQR) in the subgroup of pneumonia patients. Table S2: Lung mechanics (median, IQR) in the subgroup of responders. Figure S1: PaO_2_/FiO_2 _ratio of all patients at baseline in supine position, after 1 and 2 h of prone position (PP 1 h and PP 2 h), and after 1 and 2 h of prone position with additional upright position (PP + UP 1 h and PP + UP 2 h). *, ¶Statistically significant differences. Click here for file

## References

[B1] Erickson SE Martin GS Davis JL Matthay MA Eisner MD Recent trends in acute lung injury mortality: 1996-2005 Crit Care Med 2009 37 1574 1579 10.1097/CCM.0b013e31819fefdf 19325464 PMC2696257

[B2] Frutos-Vivar F Nin N Esteban A Epidemiology of acute lung injury and acute respiratory distress syndrome Curr Opin Crit Care 2004 10 1 6 15166842 10.1097/00075198-200402000-00001

[B3] Goss CH Brower RG Hudson LD Rubenfeld GD Incidence of acute lung injury in the United States Crit Care Med 2003 31 1607 1611 10.1097/01.CCM.0000063475.65751.1D 12794394

[B4] Rouby JJ Puybasset L Nieszkowska A Lu Q Acute respiratory distress syndrome: lessons from computed tomography of the whole lung Crit Care Med 2003 31 S285 S295 10.1097/01.CCM.0000057905.74813.BC 12682454

[B5] Suter PM Reducing ventilator-induced lung injury and other organ injury by the prone position Crit Care 2006 10 139 10.1186/cc4898 16677405 PMC1550882

[B6] Pelosi P Tubiolo D Mascheroni D Vicardi P Crotti S Valenza F Gattinoni L Effects of the prone position on respiratory mechanics and gas exchange during acute lung injury Am J Respir Crit Care Med 1998 157 387 393 9476848 10.1164/ajrccm.157.2.97-04023

[B7] Mentzelopoulos SD Roussos C Zakynthinos SG Prone position reduces lung stress and strain in severe acute respiratory distress syndrome Eur Respir J 2005 25 534 544 10.1183/09031936.05.00105804 15738300

[B8] Hoste EA Roosens CD Bracke S Decruyenaere JM Benoit DD Vandewoude KH Colardyn FA Acute effects of upright position on gas exchange in patients with acute respiratory distress syndrome J Intensive Care Med 2005 20 43 49 10.1177/0885066604271616 15665259

[B9] Richard JC Maggiore SM Mancebo J Lemaire F Jonson B Brochard L Effects of vertical positioning on gas exchange and lung volumes in acute respiratory distress syndrome Intensive Care Med 2006 32 1623 1626 10.1007/s00134-006-0299-y 16896856

[B10] Bernard GR Artigas A Brigham KL Carlet J Falke K Hudson L Lamy M Legall JR Morris A Spragg R The American-European Consensus Conference on ARDS. Definitions, mechanisms, relevant outcomes, and clinical trial coordination Am J Respir Crit Care Med 1994 149 818 824 7509706 10.1164/ajrccm.149.3.7509706

[B11] McAuley DF Giles S Fichter H Perkins GD Gao F What is the optimal duration of ventilation in the prone position in acute lung injury and acute respiratory distress syndrome? Intensive Care Med 2002 28 414 418 10.1007/s00134-002-1248-z 11967594

[B12] Taccone P Pesenti A Latini R Polli F Vagginelli F Mietto C Caspani L Raimondi F Bordone G Iapichino G Mancebo J Guerin C Ayzac L Blanch L Fumagalli R Tognoni G Gattinoni L Prone positioning in patients with moderate and severe acute respiratory distress syndrome: a randomized controlled trial JAMA 2009 302 1977 1984 10.1001/jama.2009.1614 19903918

[B13] Gattinoni L Tognoni G Pesenti A Taccone P Mascheroni D Labarta V Malacrida R Di Giulio P Fumagalli R Pelosi P Brazzi L Latini R Effect of prone positioning on the survival of patients with acute respiratory failure N Engl J Med 2001 345 568 573 10.1056/NEJMoa010043 11529210

[B14] Guerin C Gaillard S Lemasson S Ayzac L Girard R Beuret P Palmier B Le QV Sirodot M Rosselli S Cadiergue V Sainty JM Barbe P Combourieu E Debatty D Rouffineau J Ezingeard E Millet O Guelon D Rodriguez L Martin O Renault A Sibille JP Kaidomar M Effects of systematic prone positioning in hypoxemic acute respiratory failure: a randomized controlled trial JAMA 2004 292 2379 2387 10.1001/jama.292.19.2379 15547166

[B15] Sud S Friedrich JO Taccone P Polli F Adhikari NK Latini R Pesenti A Guerin C Mancebo J Curley MA Fernandez R Chan MC Beuret P Voggenreiter G Sud M Tognoni G Gattinoni L Prone ventilation reduces mortality in patients with acute respiratory failure and severe hypoxemia: systematic review and meta-analysis Intensive Care Med 2010 36 585 599 10.1007/s00134-009-1748-1 20130832

[B16] The Acute Respiratory Distress Syndrome Network Ventilation with lower tidal volumes as compared with traditional tidal volumes for acute lung injury and the acute respiratory distress syndrome N Engl J Med 2000 342 1301 1308 10793162 10.1056/NEJM200005043421801

[B17] Gattinoni L Vagginelli F Carlesso E Taccone P Conte V Chiumello D Valenza F Caironi P Pesenti A Decrease in PaCO_2 _with prone position is predictive of improved outcome in acute respiratory distress syndrome Crit Care Med 2003 31 2727 2733 10.1097/01.CCM.0000098032.34052.F9 14668608

[B18] Hager DN Krishnan JA Hayden DL Brower RG Tidal volume reduction in patients with acute lung injury when plateau pressures are not high Am J Respir Crit Care Med 2005 172 1241 1245 10.1164/rccm.200501-048CP 16081547 PMC2718413

[B19] Broccard AF Shapiro RS Schmitz LL Ravenscraft SA Marini JJ Influence of prone position on the extent and distribution of lung injury in a high tidal volume oleic acid model of acute respiratory distress syndrome Crit Care Med 1997 25 16 27 10.1097/00003246-199701000-00007 8989171

[B20] Valenza F Guglielmi M Maffioletti M Tedesco C Maccagni P Fossali T Aletti G Porro GA Irace M Carlesso E Carboni N Lazzerini M Gattinoni L Prone position delays the progression of ventilator-induced lung injury in rats: does lung strain distribution play a role? Crit Care Med 2005 33 361 367 10.1097/01.CCM.0000150660.45376.7C 15699840

[B21] Klingstedt C Hedenstierna G Lundquist H Strandberg A Tokics L Brismar B The influence of body position and differential ventilation on lung dimensions and atelectasis formation in anaesthetized man Acta Anaesthesiol Scand 1990 34 315 322 10.1111/j.1399-6576.1990.tb03094.x 2188475

[B22] Jones AT Hansell DM Evans TW Pulmonary perfusion in supine and prone positions: an electron-beam computed tomography study J Appl Physiol 2001 90 1342 1348 10.1063/1.1376404 11247933

[B23] Nyren S Radell P Lindahl SG Mure M Petersson J Larsson SA Jacobsson H Sanchez-Crespo A Lung ventilation and perfusion in prone and supine postures with reference to anesthetized and mechanically ventilated healthy volunteers Anesthesiology 2010 112 682 687 10.1097/ALN.0b013e3181cf40c8 20179506

