# Supplementary material for: Short-term effects of combining upright and prone positions in patients with ARDS: a prospective randomized study
Source: Crit Care. 2011 Sep 29;15(5):R230. doi: 10.1186/cc10471 (PMC3334777; doi:10.1186/cc10471)
Supplement: Additional file 1 — Additional information. Table S1: Main results (median, IQR) in the subgroup of pneumonia patients. Table S2: Lung mechanics (median, IQR) in the subgroup of responders. Figure S1: PaO2/FiO2 ratio of all patients at baseline in supine position, after 1 and 2 h of prone position (PP 1 h and PP 2 h), and after 1 and 2 h of prone position with additional upright position (PP + UP 1 h and PP + UP 2 h). *, ¶Statistically significant differences. [file cc10471-S1.DOC]

Table 1: Main results (Median, IQR) in the subgroup of pneumonia patients (n=16, group A: n=7, group B: n=9)

| **All patients** | **Supine** | **PP 1h** | **PP 2h** | **PP + UP 1h** | **PP + UP 2h** |  |
| --- | --- | --- | --- | --- | --- | --- |
| PaO_2_/FiO_2_ | 131 (107-169)* | 161 (131-178) | 164 (134-177) | 152 (119-177) | 189 (146-208)* |  |
| PaCO_2_ | 59 (46-68) | 60 (50-67) | 58 (48-65) | 61 (52-69) | 59 (53-69) |  |
| C_tot_ | 28.0 (22.5-42.8) | 30.5 (24.0-38.5) | 30.0 (25.0-39.3) | 28.5 (26.0-34.5) | 28.0 (22.5-34.5) |  |
| C_pulm_ | 54.5 (37.3-82.5) | 50.0 (40.5-74.8) | 57.0 (38.8-71.5) | 49.0 (39.3-71.8) | 50.5 (42.3-70.0) |  |
| C_cw_ | 87.0 (53.0-148.0) | 86.5 (52.3-118.3) | 91.5 (46.0-125.0) | 84.5 (44.5-118.5) | 75.0 (40.5-105.3) |  |
|  |  |  |  |  |  |  |
| **Group A** | **Supine** | **PP 1h** | **PP 2h** | **PP + UP 1h** | **PP + UP 2h** | **PP + UP 6h** |
| PaO_2_/FiO_2_ | 169 (118-247)^‡^ | 172 (157-253) | 170 (157-274) | 175 (163-237) | 193 (169-269)^‡^ | 242 (186-291) |
| PaCO_2_ | 62 (44-66) | 64 (57-65) | 62 (48-65) | 65 (56-69) | 62 (53-67) | 63 (41-70) |
| C_tot_ | 28.0 (24.0-42.0) | 32.0 (24.0-42.0) | 30.0 (16.0-45.0) | 30.0 (27.0-38.0) | 29.0 (28.0-39.0) | 36.0 (31.0-47.0) |
| C_pulm_ | 65.0 (53.0-83.0) | 67.0 (52.0-88.0) | 64.0 (59.0-88.0) | 57.0 (49.0-74.0) | 56.0 (53.0-84.0) | 66.0 (56.0-91.0) |
| C_cw_ | 87.0 (34.0-202.0) | 65.0 (39.0-129.0) | 58.0 (19.0-143.0) | 65.0 (41.0-123.0) | 59.0 (40.0-132.0) | 79.0 (47.0-116.0) |
|  |  |  |  |  |  |  |
| **Group B** | **Supine** | **PP + UP 1h** | **PP + UP 2h** | **PP 1h** | **PP 2h** | **PP 6h** |
| PaO_2_/FiO_2_ | 128 (82-146)^§^ | 120 (118-152) ^II^ | 184 (126-196)^§II^ | 138 (116-171) | 142 (120-171) | 144 (113-180) |
| PaCO_2_ | 49 (47-70) | 57 (50-70) | 56 (52-73) | 53 (49-69) | 51 (48-65) | 58 (53-70) |
| C_tot_ | 26.0 (20.5-43.0) | 28.0 (24.5-31.5) | 27.0 (21.0-31.5) | 30.0 (24.0-38.0) | 31.0 (25.0-38.5) | 30.0 (23.5-35.5) |
| C_pulm_ | 45.0 (29.5-82.0) | 40.0 (31.5-63.5) | 43.0 (31.5-60.0) | 44.0 (34.0-61.5) | 44.0 (32.0-64.0) | 44.0 (28.5-55.0) |
| C_cw_ | 87.0 (66.0-131.0) | 99.0 (60.5-114.0) | 76.0 (50.0-99.0) | 93.0 (65.0-116.5) | 95.0 (68.5-109.0) | 105.0 (58.5-153.0) |
|  |  |  |  |  |  |  |

* Supine v.s. PP + UP 2 hours p<0.001

‡ Supine v.s. PP + UP 2 hours, p<0.05

§ Supine v.s. PP + UP 2 hours, p<0.001

II PP+ UP 2 hours v.s. PP 1 hour, p<0.05

Table 2: Lung mechanics (Median, IQR) in the subgroup of responders (n=14, group A: n=7, group B: n=7)

| **All patients** | **Supine** | **PP 1h** | **PP 2h** | **PP + UP 1h** | **PP + UP 2h** |  |
| --- | --- | --- | --- | --- | --- | --- |
| C_tot_ | 28.5 (23.5-37.0) | 30.5 (23.8-36.3) | 30.5 (22.8-37.0) | 28.5 (25.5-32.3) | 28.0 (23.8-32.8) |  |
| C_pulm_ | 53.5 (38.3-81.5) | 48.0 (39.3-78.3) | 53.5 (39.5-76.8) | 46.5 (35.5-75.8) | 48.0 (40.5-72.8) |  |
| C_cw_ | 72.5 (50.8-87.5) | 78.0 (51.3-96.0) | 73.5 (51.8-89.8) | 70.0 (47.5-101.3) | 73.5 (41.5-86.3) |  |
|  |  |  |  |  |  |  |
| **Group A** | **Supine** | **PP 1h** | **PP 2h** | **PP + UP 1h** | **PP + UP 2h** | **PP + UP 6h** |
| C_tot_ | 28.0 (22.0-31.0) | 30.0 (20.0-32.0) | 30.0 (19.0-31.0) | 28.0 (27.0-30.0) | 28.0 (27.0-32.0) | 34.0 (31.0-36.0) |
| C_pulm_ | 51.0 (39.0-77.0) | 49.0 (44.0-88.0) | 52.0 (44.0-88.0) | 47.0 (42.0-74.0) | 48.0 (43.0-84.0) | 66.0 (43.0-91.0) |
| C_cw_ | 67.0 (44.0-87.0) | 65.0 (39.0-92.0) | 58.0 (39.0-95.0) | 65.0 (43.0-100.0) | 59.0 (42.0-96.0) | 79.0 (47.0-111.0) |
|  |  |  |  |  |  |  |
| **Group B** | **Supine** | **PP + UP 1h** | **PP + UP 2h** | **PP 1h** | **PP 2h** | **PP 6h** |
| C_tot_ | 35.0 (24.0-43.0) | 36.0 (25.0-39.0) | 37.0 (24.0-40.0) | 29.0 (24.0-37.0) | 27.0 (20.0-37.0) | 33.0 (23.0-36.0) |
| C_pulm_ | 64.0 (36.0-83.0) | 47.0 (37.0-75.0) | 55.0 (35.0-73.0) | 40.0 (37.0-81.0) | 45.0 (36.0-72.0) | 54.0 (32.0-64.0) |
| C_cw_ | 73.0 (59.0-89.0) | 79.0 (65.0-113.0) | 76.0 (66.0-88.0) | 78.0 (49.0-105.0) | 76.0 (27.0-83.0) | 80.0 (47.0-82.0) |
|  |  |  |  |  |  |  |

Figure 1: PaO_2_/FiO_2_ – ratio of all patients at baseline in supine position, after 1 and 2 hours of prone position (PP 1h and PP 2h), and after 1 and 2 hours of prone position with additional upright position (PP+UP 1h and PP+UP 2h). Asterisks and paragraphs denote statistically significant difference.
